# The acute adaptation of skin microcirculatory perfusion *in vivo* does not involve a local response but rather a centrally mediated adaptive reflex

**DOI:** 10.3389/fphys.2023.1177583

**Published:** 2023-05-04

**Authors:** Luís Monteiro Rodrigues, Clemente Rocha, Sérgio Andrade, Tiago Granja, João Gregório

**Affiliations:** CBIOS—Universidade Lusofona’s Research Center for Biosciences and Health Technologies, Universidade Lusófona (Lisbon’s University Campus), Lisbon, Portugal

**Keywords:** venoarteriolar reflex, reactive hyperemia, acute perfusion adaptation, cardiovascular homeostasis, skin microcirculation, local and central reflexes

## Abstract

**Introduction:** Cardiovascular homeostasis involves the interaction of multiple players to ensure a permanent adaptation to each organ’s needs. Our previous research suggested that changes in skin microcirculation—even if slight and distal—always evoke an immediate global rather than “local” response affecting hemodynamic homeostasis. These observations question our understanding of known reflexes used to explore vascular physiology, such as reactive hyperemia and the venoarteriolar reflex (VAR). Thus, our study was designed to further explore these responses in older healthy adults of both sexes and to potentially provide objective evidence of a centrally mediated mechanism governing each of these adaptive processes.

**Methods:** Participants (n = 22, 52.5 ± 6.2 years old) of both sexes were previously selected. Perfusion was recorded in both feet by laser Doppler flowmetry (LDF) and photoplethysmography (PPG). Two different maneuvers with opposite impacts on perfusion were applied as challengers to single limb reactive hyperemia evoked by massage and a single leg pending to generate a VAR. Measurements were taken at baseline (Phase I), during challenge (Phase II), and recovery (Phase III). A 95% confidence level was adopted. As proof of concept, six additional young healthy women were selected to provide video imaging by using optoacoustic tomography (OAT) of suprasystolic post-occlusive reactive hyperemia (PORH) in the upper limb.

**Results:** Modified perfusion was detected by LDF and PPG in both limbs with both hyperemia and VAR, with clear systemic hemodynamic changes in all participants. Comparison with data obtained under the same conditions in a younger cohort, previously published by our group, revealed that results were not statistically different between the groups.

**Discussion:** The OAT documentary and analysis showed that the suprasystolic pressure in the arm changed vasomotion in the forearm, displacing blood from the superficial to the deeper plexus vessels. Deflation allowed the blood to return and to be distributed in both plexuses. These responses were present in all individuals independent of their age. They appeared to be determined by the need to re-establish hemodynamics acutely modified by the challenger, which means that they were centrally mediated. Therefore, a new mechanistic interpretation of these exploratory maneuvers is required to better characterize in vivo cardiovascular physiology in humans.

## 1 Introduction

Cardio-circulatory adaptation is essential in managing acute physiological challenges (e.g., posture change) as in chronic pathological processes (e.g., hypertension or heart failure) to continuously harmonize hemodynamics with cell needs. A permanent crosstalk between microcirculation and higher circulatory structures involving different sensors and effectors, central and peripheral, is part of these complex mechanisms (Mazzoni & Schmid-Schonbein, 1996; Mei et al., 2018; Cracowski & Roustit, 2020; Guven et al., 2020). However, the details of these interactions remain largely unknown.

In normal physiology, acute perfusion adaptations, such as those originating from sudden changes in venous and capillary flow and pressure are believed to be mostly determined by tissue-related intervenients (neuro-humoral, metabolic, and endocrine) converging to a “local reflex” thought to protect the microcirculatory unit (Rowell et al., 2011; Masashi et al., 2013). The venoarteriolar reflex (VAR) (Cracowski & Roustit 2020; Low 2004; Silva et al., 2018) is one well-known example. In the opposite direction, sudden perfusion changes due to imposed suprasystolic pressure applied to a main artery's post-occlusive reactive hyperemia (PORH) have also been explained on the basis of local endothelial vasodilators’ sensory nerves and myogenic activities (Cracowski & Roustit 2020; Rosenberry & Nelson MD 2020). No matter their limitations, non-agreement regarding the character of the mechanisms involved, VAR and PORH have been long used in experimental physiology to provide a functional window to assess vascular physiology in human (Cracowski & Roustit 2020; Low 2004; Silva et al., 2018; Rosenberry & Nelson MD 2020; Crandall et al., 2002). Nevertheless, recent studies on these cardio-circulatory responses suggest a different view.

This localized reactive hyperemia evoked by short-term low-intensity massage in one of the lower limbs produced a statistically significant observable perfusion increase in the contralateral non-massaged limb in humans (Rocha C et al., 2018a; Rocha C et al., 2018b; Rodrigues et al., 2020). Similar observations were recorded in the upper limb with a suprasystolic pressure cuff maneuver (Florindo et al., 2021; Monteiro Rodrigues et al., 2022). In addition, the classical VAR evoked a perfusion reduction in the contralateral resting limb (Cracowski & Roustit 2020; Silva et al., 2018; Crandall et al., 2002). All these observations strongly suggested that any modification of local perfusion produced an immediate integrative parallel response that was proportional to the challenge intensity to restore hemodynamics. We named it a prompt adaptive hemodynamic response (PAHR) and considered its potential utility as a global marker with clinical relevance to further characterize *in vivo* cardiovascular physiology. Apart from these observations, we recognized that this potential endpoint is insufficiently characterized, especially regarding common determinants such as age, and that further evidence of the existence of this supposed centrally mediated reflex is still required.

To these purposes, the present study was designed to extend some previously published results (Silva et al., 2015; 2018; Rodrigues et al., 2020) to study these responses to two challengers—superficial hyperemia (massage) and the venoarteriolar reflex in an older group of healthy participants. In complement and as a part of the “proof of concept,” the study included the use of functional imaging to create a mechanistic documentary of a PORH maneuver in the upper limb during a suprasystolic pressure cuff experiment.

## 2 Materials and methods

For the present study, a convenience sample of 22 healthy individuals (52.5 ± 6.2 years old) of both sexes (*n* = 11 each) was selected after informed written consent (Group A). Specific inclusion/non-inclusion criteria that were previously defined for a similar research (Rodrigues et al., 2020) were applied. All participants were normotensive with no signs of vascular impairment, confirmed by the ankle–brachial index (ABI, 1.1 ± 0.1) (Aboyans et al., 2013), normal body mass index (BMI, 24.8 ± 2.1), and (self-reported) equivalent levels of physical activity. Furthermore, participants were non-smokers and free of any regular medication or food supplementation. All female participants were menopausal without hormone-replacement therapy. Study participants were asked to refrain from consuming caffeinated and/or any other vasoactive beverages for 24 h prior to the experiments. Table 1 summarizes the general characteristics of these participants.

**TABLE 1:** Participants’ characterization. Results are presented as medians and Q1–Q3 (25th empirical quartile to 75th empirical quartile). Statistical comparison between sexes within the age group using the Mann–Whitney test (*p < 0.05).

	Men	Women	*p*
N (%)	11 (50)	11 (50)	—
Smokers (%)	No (100)	No (100)	—
Age, years (Q1–Q3)	53.0 (46.0–58.0)	51.0 (48.5–54.5)	0.921
Body mass, kg (Q1–Q3)	76.0 (74.5–81.5)	65.0 (60.0–68.3)	0.002*
Height, m (Q1–Q3)	1.75 (1.72–1.81)	1.60 (1.56–1.64)	<0.001*
BMI, kg/m^2^ (Q1–Q3)	25.4 (24.4–25.8)	24.8 (22.8–26.3)	0.949
SYSTP, mmHg (Q1–Q3)	124.7 (117.8–127.0)	114.0 (112.3–131.8)	0.606
DIASP, mmHg (Q1–Q3)	84.0 (76.6–88.0)	77.5 (76.0–78.2)	0.743
ABI (Q1–Q3)	1.17 (1.06–1.17)	1.14 (1.06–1.16)	0.148
PR, bpm (Q1–Q3)	57.0 (52.0–63.8)	64.0 (59.7–68.3)	0.094

BMI, body mass index; SYSTP, systolic pressure; DIASP, diastolic pressure; ABI, ankle–brachial index; PR, pulse rate; bpm, beats per minute.

An additional group of six young healthy women (mean age, 23.0 ± 2.19 years) using similar health selection criteria was chosen to document the circulatory mechanisms involved in the PORH maneuver using functional imaging (Group B).

All procedures were previously approved by the institutional ethical commission and carried out in accordance with the Declaration of Helsinki and its respective amendments, observing good clinical practices for medical research involving human participants (WMA 2013).

### 2.1 Experimental

Experiments took place with controlled temperature, humidity (21°C ± 1°C; 40%–60%), and light. Participants were acclimatized to room conditions for approximately 20 min while lying comfortably in the supine position.

#### 2.1.1 Hyperemia

Participants in Group A (*n* = 22) had one randomly chosen leg massaged, while the contralateral leg (in the same position) served as the control. This procedure, applied by a licensed, experienced therapist, involved the application of short, rhythmic, light repetitive strokes in the ascending direction from the ankle to the knee, with the palms of both hands gliding along one (challenged) leg’s longitudinal axis (Rocha et al., 2016). The protocol included three phases—a 10-min baseline resting register (Phase I), a 5-min massage challenge (Phase II), and a 10-min recovery (Phase III).

#### 2.1.2 Venoarteriolar reflex

From the participant pool of Group A, five men and five women (mean age, 50.3 ± 5.64 years; ABI, 1.1 ± 0.1; and BMI, 24.9 ± 2.2) volunteered to participate in a standard VAR procedure (Low 2004; Rathbun et al., 2008). The experimental procedure likewise involved a baseline recording in the supine position with both legs lying parallel to the body axis (Phase I), the challenge with one randomly chosen leg pending approximately 50 cm below the heart level (Phase II), and recovery (Phase III), resuming the initial position.

#### 2.1.3 Functional imaging: proof of concept

To document the perfusion changes in these conditions, a classical post-occlusive reactive hyperemia (PORH) maneuver was applied to Group B participants (*n* = 6) and videos were recorded by optoacoustic tomography (OAT) for further analysis. A pressure cuff was applied in the middle of the upper arm. After stabilization (Phase I), the cuff was rapidly inflated to 200 mmHg to occlude the brachial artery for approximately 1 min to ensure hemodynamical stabilization in the area (Phase II). The cuff was then deflated for recovery (Phase III).

### 2.2 Measurement technologies and signal processing

For the massage and VAR protocols, blood perfusion was continuously assessed through laser Doppler flowmetry (LDF) and photoplethysmography (PPG), two optical technologies based on related principles but with different skin depth capacities (Ash et al., 2017; Bergstrand et al., 2009; Rodrigues et al., 2019). LDF and PPG sensors were applied in the plantar aspects of the second and first toes, respectively, in both feet. This strategy substantially reduces variability when measuring in the distal inferior limb (Silva et al., 2018; Rodrigues et al., 2020; Rodrigues et al., 2018). The LDF signal, expressed in arbitrary blood perfusion units (BPUs), was obtained with a Perimed PF 5010 (Perimed, Sweden) system with a pair of P457 probes secured with a PF 105-3 tape. The skin surface temperature was also continuously monitored by the system. The PPG signal, expressed in BPUs, was obtained by a BITalino Plugged Kit (PLUX Biosignals, Portugal), which also recorded the blood volume pulse (BVP) and heart rate. The blood pressure (systolic and diastolic) was measured in the arm (Tensoval Comfort, Hartman, Germany) in each procedural phase.

For functional imaging, we used an optoacoustic system from iThera Medical GmbH (Munich, Germany) MSOT for multi-spectral optoacoustic tomography. This technology detects sound waves generated by molecules previously excited by light from a wave laser beam. In consequence, a transitory thermoelastic expansion occurs that characterizes the specific (excited) chromophores. The signal pattern across the measured wavelengths serves as an exclusive absorption feature for each (chromophore) molecule (Daiwei et al., 2021; Hu and Wang 2010; Wang 2008). Recent specifications and operation details of this technology are available elsewhere (Daiwei et al., 2021; Granja et al., 2022; Hu and Wang 2010; Monteiro Rodrigues et al., 2022; Wang 2008).

OAT measurements were taken in the same limb, in the volar forearm, with the measurement probe held in contact with the skin surface using a flexible metal arm (Granja et al., 2022). The system allows the visualization of the chromophores from oxyhemoglobin HbO2 and deoxyhemoglobin Hb in real time during the acquisition. Recorded videos were post-processed for image reconstruction by the viewMSOT software (iThera Medical, version 4.0), which allows the quantification of HbO2, Hb, total hemoglobin (HbT as the sum of HbO_2_ and Hb), and the mean saturation of oxygen (mSO2). The ImageJ software (National Institutes of Health, version 1.53k14) was also used in the image reconstruction process.

### 2.3 Statistical analysis

Descriptive and comparative statistics were applied to LDF and PPG signals using the IBM SPSS v 22.0 software (IBM Corporation, NY, United States) and jamovi software version 2.2 (jamovi project, Sydney, AU). Perfusion changes were calculated as the mean value of the LDF area under the curve and as the mean amplitude of the PPG signal. Upon confirmation of normal data distribution by the Shapiro–Wilk test, parametric (repeated measures ANOVA with the Tukey test for *post hoc* correction) or non-parametric tests (Kruskal–Wallis and Friedman test with paired comparison corrections) were chosen. For the functional imaging analysis, we applied one-way ANOVA with Tukey’s multiple comparison test with a single pooled variance distribution. A confidence level of 95% (*p* < 0.05) was assumed.

## 3 Results

**FIGURE 1:**
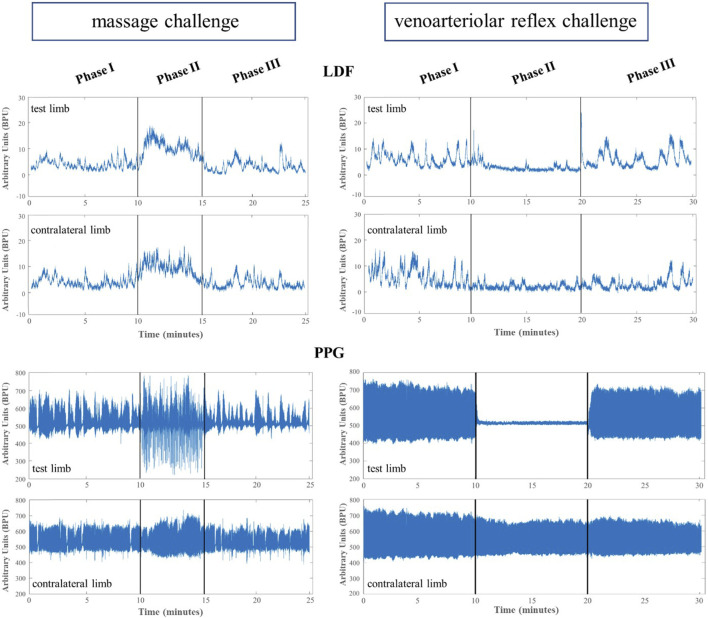
Illustrative example of the mean lower limb perfusion changes expressed in arbitrary blood perfusion units obtained by laser Doppler flowmetry (LDF) and reflection photoplethysmography (PPG) in participants’ test and contralateral lower limbs after evoking a massage hyperemia (left panels) and the venoarteriolar reflex responses (right panels).

Illustrative records of our observations are shown in Figure 1. Perfusion changes were recorded with LDF and PPG systems in both limbs during all phases of both the procedures.

The application of massage to a single limb produced a reactive hyperemia with perfusion elevation in both limbs (Table 2). This increase was statistically significant in the challenged limb foot (Phase II–Phase I), disappearing in recovery (Phase III–Phase I). The blood pressure and pulse clearly changed throughout the experimental procedure; however, the reduction in Phase II was statistically significant only for the diastolic pressure (Table 2).

**TABLE 2:** Mean perfusion changes expressed in arbitrary blood perfusion units (BPUs) obtained by laser Doppler flowmetry (LDF) and reflection photoplethysmography (PPG) in aged adult participants’ test and contralateral feet after application of the massage procedure (see text). Blood pressure (systolic and diastolic, mm Hg) and pulse (bpm) are also shown. Data are expressed as means + SD. Statistical comparison with the Friedman test with paired comparison correction (Durbin–Conover) between lower limbs across phases. The blood pressure and pulse rate comparison assessed with repeated measures ANOVA with the Tukey test for *post hoc* correction. *p < 0.05.

	Phase I		Phase II		Phase III		Phase II–Phase I (*p*-value)		Phase III–Phase I (*p*-value)		All phases
	Test limb	Contralateral limb	Test limb	Contralateral limb	Test limb	Contralateral limb	Test limb	Contralateral limb	Test limb	Contralateral limb	
LDF (BPUs)	19.6 + 27.2	25.8 + 45.8	35.2 + 43.3	30.6 + 49.0	23.3 + 31.7	23.4 + 37.2					
*p*-value	0.079		0.049*		0.518		<0.001*	0.518	0.145	0.344	0.002*
PPG (BPUs)	148.7 + 141.3	138.5 + 129.9	208.8 + 152.8	156.8 + 137.3	147.8 + 128.6	129.6 + 131.7					
*p*-value	0.554		0.054		0.612		0.036*	0.447	0.272	0.311	0.009*
	Phase I		Phase II		Phase III		Phase II–Phase I (*p*-value)		Phase III–Phase I (*p*-value)		All phases
Systolic pressure (mmHg)	121.5 + 12.5		119.5 + 12.8		123.0 + 12.1		0.116		0.332		0.034*
Diastolic pressure (mmHg)	80.3 + 7.4		77.2 + 5.3		81.0 + 7.1		0.002*		0.679		<0.001*
Pulse rate (bpm)	62.0 + 10.7		60.4 + 10.8		61.9 + 11.7		0.185		0.992		0.098

A comparison of LDF and PPG data obtained from all participants for each phase of the experimental procedures (Table 2) revealed perfusion differences between limb pairs in Phase II, but not in Phases I and III. These perfusion differences, detected with both LDF and PPG, could only be attributed to the challenge.

The VAR procedure evoked a significant reduction in perfusion in Phase II with both LDF and PPG in the pending limb and in the contralateral (resting) limb (Table 3). Changes in blood pressure were also detected, but significant differences could not be found. After resuming the initial position, perfusion recovered in both limbs, although at the end of Phase III, perfusion remained lower than at baseline, indicating a slower establishment of a new homeostatic state.

**TABLE 3:** Mean perfusion changes expressed in arbitrary blood perfusion units (BPUs) obtained by laser Doppler flowmetry (LDF) and reflection photoplethysmography (PPG) in aged adults participants’ test and contralateral feet after the single leg dropping procedure to evoke the venoarteriolar reflex (see text). Data are expressed as means + SD. Statistical comparison with the Friedman test with paired comparison correction (Durbin–Conover) between feet across phases. *p < 0.05.

	Phase I		Phase II		Phase III		Phase II–Phase I (*p*-value)		Phase III–Phase I (*p*-value)		All phases
	Test limb	Control limb	Test limb	Control limb	Test limb	Control limb	Test limb	Control limb	Test limb	Control limb	
LDF (BPUs)	49.1 ± 52.9	46.4 ± 49.3	24.1 ± 36.7	38.3 ± 48.0	39.4 ± 43.4	41.4 ± 51.2					
*p*-value	0.294		0.004		0.294		<0.001	0.028	0.547	0.547	<0.001
PPG (BPUs)	231.3 ± 194.5	167.4 ± 123.5	172,4 ± 69.3	128.8 ± 97.3	205.1 ± 199.2	140.0 ± 103.8					
*p*-value	0.521		0.019		0.630		<0.001	0.006	0.059	0.082	<0.001
	Phase I		Phase II		Phase III		Phase II–Phase I (*p*-value)		Phase III–Phase I (*p*-value)		All phases
Systolic pressure (mmHg)	119,2 + 9.6		123.6 + 11.0		125.0 + 10.4		0.086		0.422		0.078
Diastolic pressure (mmHg)	82.1 + 4.3		83,4 + 6.4		84.0 + 6.3		0.222		0.739		0.734
Pulse rate (bpm)	61.8 + 6.9		63,2 + 10.7		69.3 + 7.2		0.185		0.919		0.083

**FIGURE 2:**
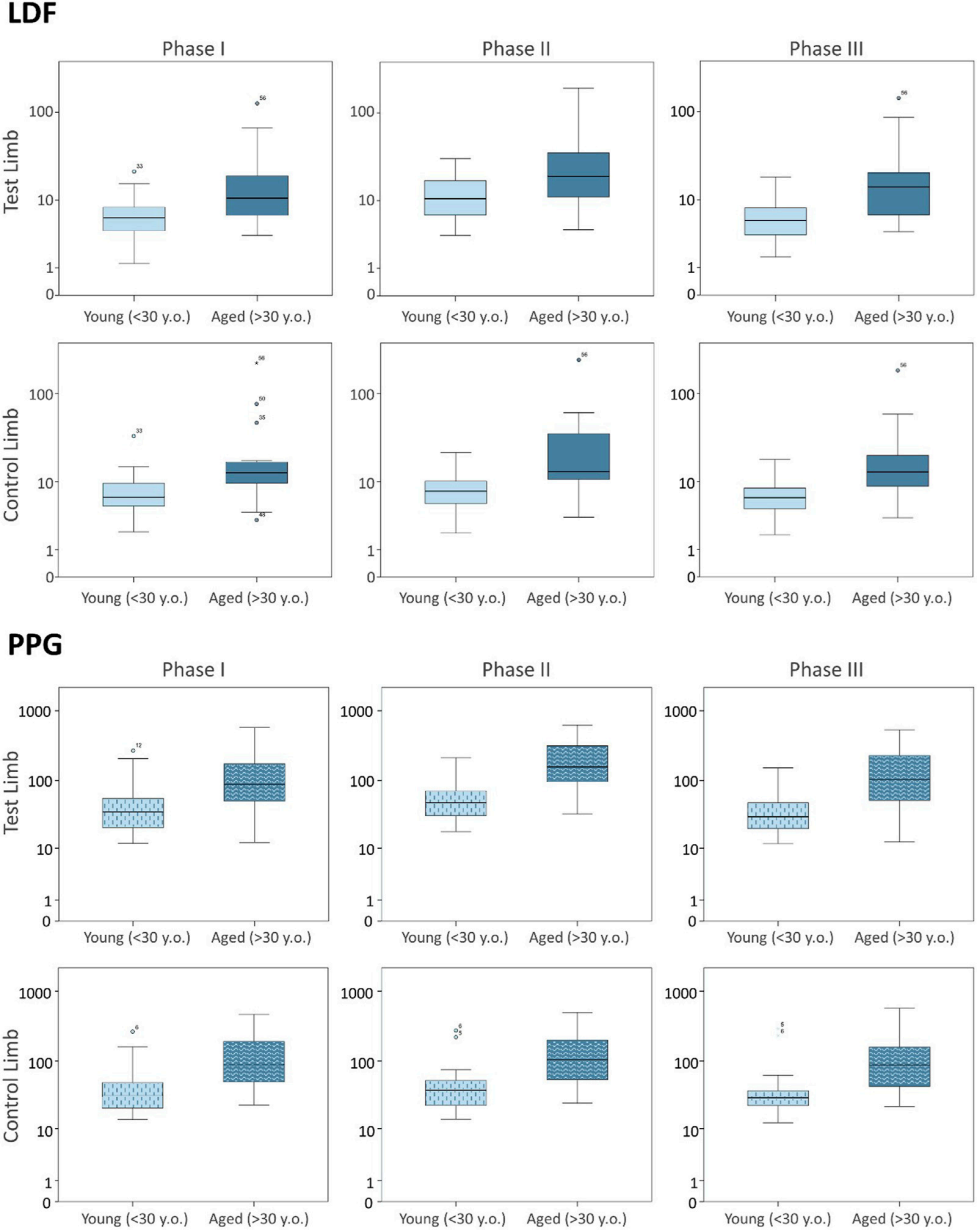
Blood perfusion changes registered as a consequence of massage hyperemia applied to one leg measured simultaneously by LDF (upper panel) and PPG (lower panel) in the test and contralateral feet in two different age groups. Data from the current age group (A) study is compared with data from a younger cohort previously published (Rodrigues et al., 2020), which is reproduced under the same protocol conditions (see section 3). The log scale is only applied to better visualize data distribution.

**FIGURE 3:**
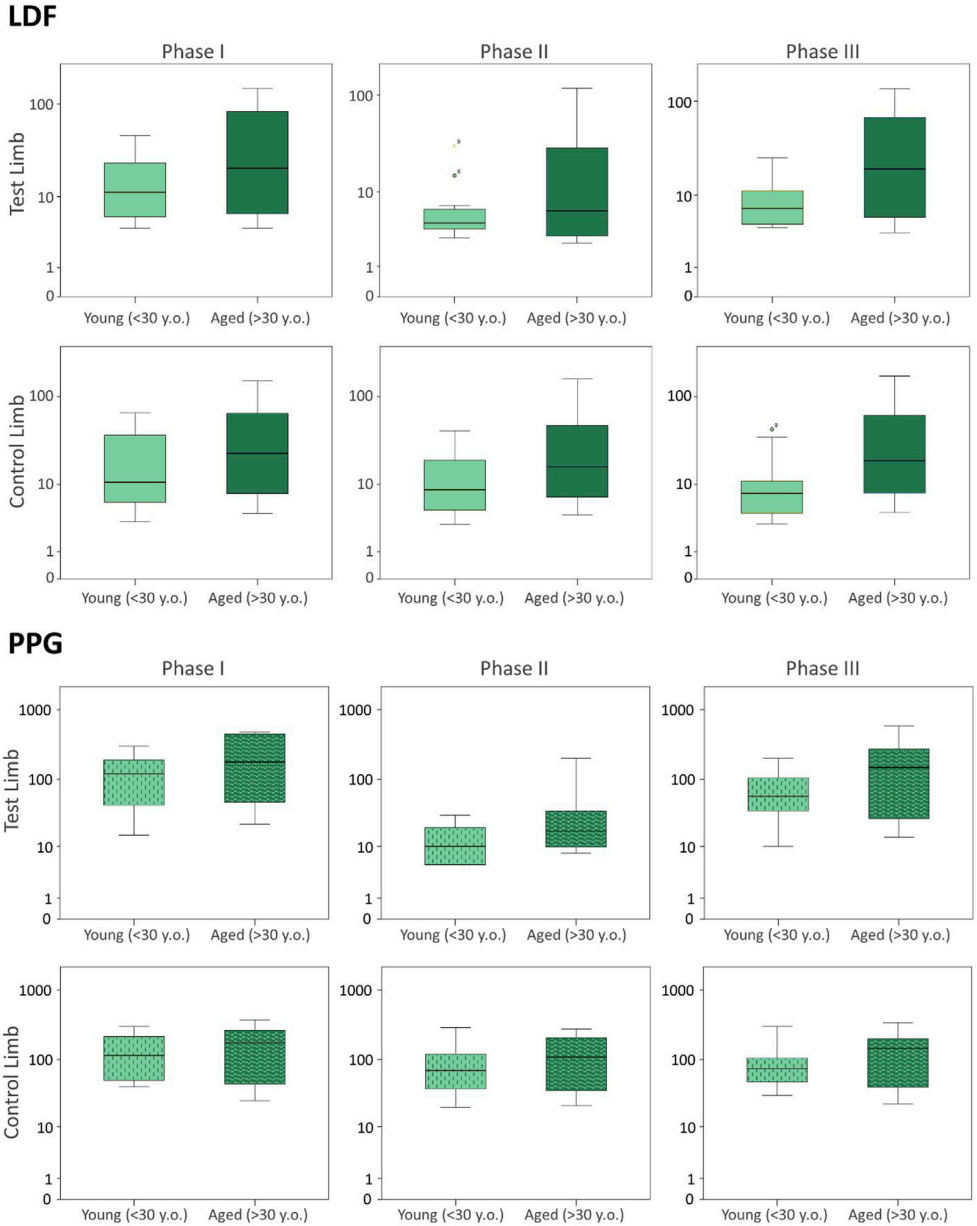
Blood perfusion changes registered as a consequence of a venoarteriolar reflex evoked in a single leg measured simultaneously by LDF (upper panel) and PPG (lower panel) in the test and contralateral feet of two different age groups. Data from the current age group (A) study is compared with data from a younger cohort previously published (Silva et al., 2018), which is reproduced under the same protocol conditions (see section 3). The log scale is applied to better visualize data distribution.

To further disclose the potential effect of age on the evoked perfusion adaptive mechanisms, the evolution of these perfusion variables was compared to data from identical experimental conditions applied to a group of young participants recently published involving massage (Rodrigues et al., 2020) and VAR (Silva et al., 2018) interventions. Figures 2, 3 display the perfusion evolution obtained in these young and aged individuals with massage–hyperemia and with the pending leg VAR procedures, respectively. Aged participants have shown significantly higher perfusions in both limbs than the younger group (both in LDF and PPG perfusion data (Figure 2). Regarding VAR, differences between age groups were not statistically significant in any protocol, with the exception of LDF perfusion data in the control limb in Phase III (*p* = 0.041). Nevertheless, the evolution profiles in both groups were similar for each protocol. Comparing the extent of these interventions, measured as the difference in percentage between each phase, we found no significant differences in the magnitude of the effect between age groups, as shown in Tables 4, 5.

**TABLE 4:** Perfusion differences (%) between Phase II and Phase I, and Phase III and Phase I, for both limbs by the age group in the massage protocol. As the assumption of normality was not met, non-parametric tests were performed. ¥—Statistical comparison between lower limbs with the Friedman test with paired comparison correction (Durbin–Conover) statistical comparison between participants of two different age groups with the Kruskal–Wallis test and the Dwass–Steel–Critchlow–Fligner procedure for pairwise comparisons of perfusion changes obtained in the same experimental conditions.

LDF	Median % Δ Phase II–Phase I (Q1–Q3)			Median % Δ Phase III–Phase I (Q1–Q3)		
	Test limb	Contralateral limb	*p*-value¥	Test limb	Contralateral limb	*p*-value¥
Young	83.6 (47.6–117.3)	7.1 (−3.3–34.0)	<0.001	−2.1 (−13.1–14.4)	−5.3 (−9.5–0.2)	0.501
Age	59.6 (23.1–88.0)	7.83 (−8.5–14.7)	<0.001	9.4 (−3.8–22.2)	−9.4 (−20.3–16.3)	0.029
*p*-value⸸	0.174	0.374		0.067	0.568	

PPG	Median % Δ Phase II–Phase I (Q1–Q3)			Median % Δ Phase III–Phase I (Q1–Q3)		
	Test limb	Contralateral limb	*p*-value¥	Test limb	Contralateral limb	*p*-value¥
Young	32.9 (2.9–69.0)	1.4 (−17.2–23.2)	<0.001	−5.9 (−22.0–8.0)	−12.3 (−21.7–1.1)	0.606
Age	26.9 (−3.7–109.1)	3.8 (−5.6–9.6)	0.028	−7.4 (−19.5–11.4)	−12.9 (−21.5–0.6)	0.403
*p*-value⸸	0.960	0.592		0.763	0.712	

**TABLE 5:** Percentage difference between Phase II and Phase I, and Phase III and Phase I, for both limbs by the age group in the VAR protocol. As the assumption of normality was met and the homogeneity of variances was also verified, repeated measures ANOVA with the post hoc Tukey test for pairwise comparisons were performed.

LDF	Median % Δ Phase II–Phase I (Q1–Q3)			Median % Δ Phase III–Phase I (Q1–Q3)		
	Mean % Δ Phase II–Phase I (SD)			Mean % Δ Phase III–Phase I (SD)		
	Test limb	Contralateral limb	*p*-value¥	Test limb	Contralateral limb	*p*-value¥
Young	−50.4 (20.1)	−26.3 (14.9)	0.002	−36.3 (23.0)	−32.5 (24.2)	0.997
Age	−51.3 (21.8)	−18.6 (18.3)	<0.001	−5.5 (37.7)	−8.2 (25.3)	1.000
*p*-value⸸	1.000	0.952		0.312	0.345	

PPG	Median % Δ Phase II–Phase I (Q1–Q3)			Median % Δ Phase III–Phase I (Q1–Q3)		
	Test limb	Contralateral limb	*p*-value¥	Test limb	Contralateral limb	*p*-value¥
Young	−84.9 (13.7)	−29.6 (17.3)	<0.001	−36.2 (19.5)	−24.2 (22.0)	0.248
Age	−77.9 (18.0)	−21.1 (14.7)	<0.001	−16.5 (23.3)	−11.4 (21.6)	0.975
*p*-value⸸	0.960	0.913		0.414	0.862	

**FIGURE 4:**
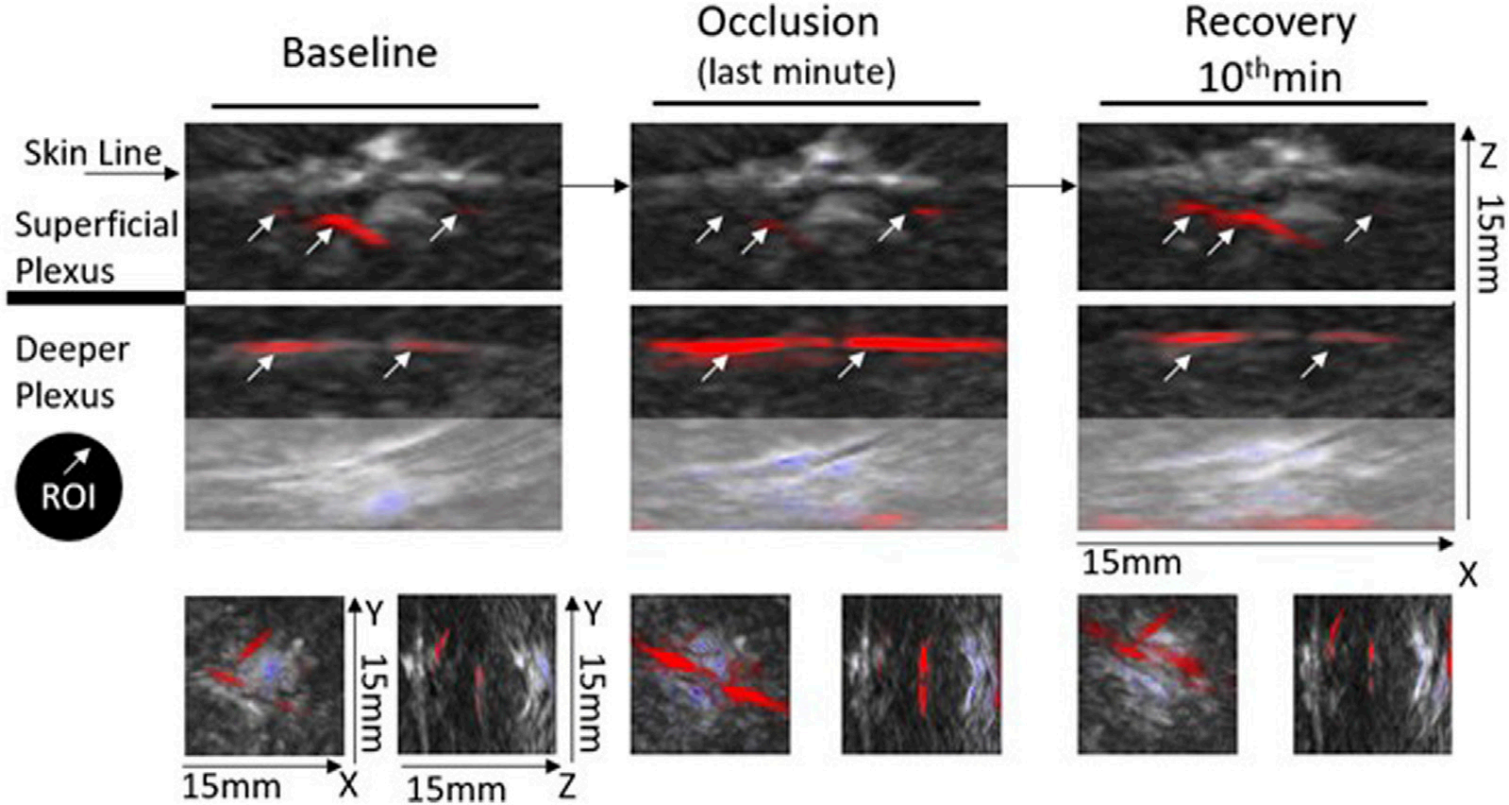
Representative optoacoustic images of a PORH maneuver in the human forearm—images were selected from continuous acquisition and displayed according to the progression of the experimental protocol. Each image section, e.g., baseline (Phase I), occlusion (Phase II), and recovery (Phase III) are depicted with a depth of 15 mm in the XYZ axis. Each image section (orthogonal view XZ) includes a frame from the first baseline minute, from the final second of occlusion (held for 1 min at 200 mmHg), and a frame from the final minute of recovery. Selected ROIs for analysis are indicated (white arrows) in the main XZ frame. For proper inter-individual calibration, ROIs must be identified at the deeper and superficial plexus during each acquisition. The XY and YZ axes highlight the 3D impact of the PORH maneuver across the protocol.

**FIGURE 5:**
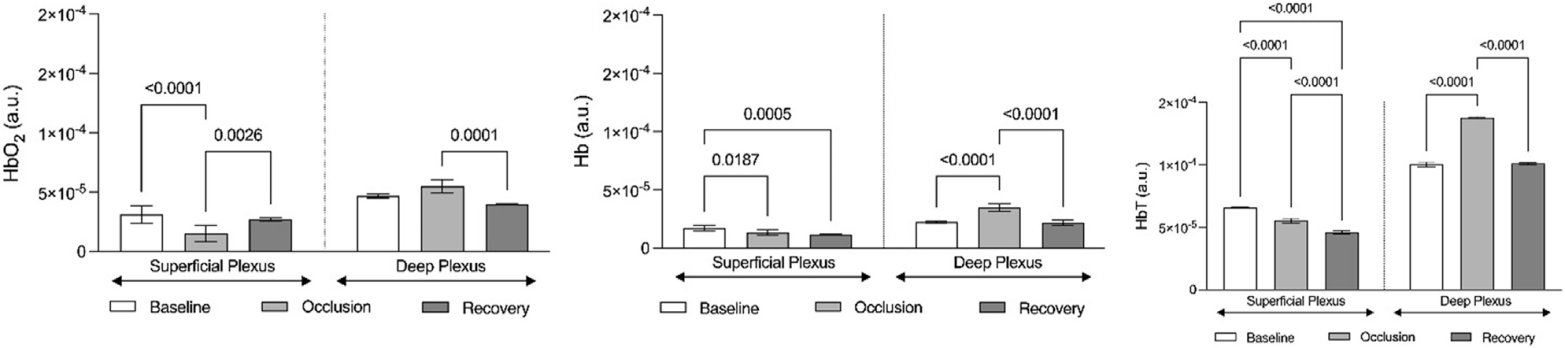
Graphic representation of the evolution of hemoglobin chromophores (HbO2, HbO, and HbT) as registered by optoacoustic tomography in the volar forearm during the course of the suprasystolic post-occlusion maneuver in the arm (*n* = 6). Hemoglobin chromophores are represented as means (+SD) at baseline (before occlusion), during occlusion, and in the recovery phase after cuff deflation. These evolutions depict the centrally mediated adaptive movement of blood between skin vascular plexus as illustrated in Figure 4 (see section 3).

The functional imaging obtained with the MSOT system during PORH revealed the skin vascular plexus parallel to the skin surface with larger deep plexus vessels, located 2–6 mm below the skin surface, and the smaller vessels and superficial plexus located 0.6–2 mm below the skin (Figure 4). Thus, we could follow in real time and full extension the effects of PORH in skin vasculature. As recently published (Monteiro Rodrigues et al., 2022), HbO2 and Hb, as quantitatively monitored by our chromophores of interest, significantly changed during the suprasystolic pressure challenge (Figures 4, 5) as a consequence of the transitory movement of blood from superficial to deep structures and back from occlusion to cuff deflation and recovery.

## 4 Discussion

Reactive hyperemia and VAR, although still poorly understood and characterized, have been widely used to explore cardiovascular physiology and pathophysiology. Our findings on the adaptive responses following reactive hyperemia and VAR in human distal limbs question earlier local mechanistic views behind their interpretation, as the effects of both maneuvers occur simultaneously in the test limb as in the contralateral limb. In other words, the intervention in one limb acutely alters perfusion in the same direction in both limbs. Once the intervention ceases, adaptive responses restore hemodynamics as part of homeostasis.

Previous studies on PORH and VAR used a variety of technologies, typically optically measuring perfusion, although in most cases, measurements were made in a single limb, at a single (contact) point. PORH and VAR observations were perceived as “local”—the hypothesis of a centrally mediated response could not be experimentally explored without the simultaneous contralateral limb measurements. In fact, these analogous responses in blood perfusion in the contralateral non-challenged limb strongly suggest that the adaptive response is centrally mediated, signifying it could be used as a marker of cardiovascular adaptation with clinical interest (Silva et al., 2018; Rodrigues et al., 2020). Therefore, recognizing that to date no clear evidence of this central mechanism had been presented and aware of the potential bias introduced by obtaining results only from young healthy populations, the present study was meant to provide further data to address these issues. For this purpose, we used the same challengers as in recent publications—the (mild) reactive hyperemia produced by massage (Rocha C et al., 2018a; Rocha C et al., 2018b; Rodrigues et al., 2020) and the intense perfusion reduction evoked by one leg pending in dorsal decubitus (Silva et al., 2018) in a group of aged participants.

As shown (Figure 1; Table 2), the superficial, light intensity massage of one lower limb evoked a reactive hyperemia visibly detected in both limbs, although perfusion differences were statistically significant only in the massaged limb. Significant reductions (*p* < 0.05) in diastolic pressure were also noted in this aged group (Table 2). To look deeper into the effect of age on the evolution of these perfusion profiles, we further compared these values with those of the younger participants obtained under the same conditions (Figure 2) (Rodrigues et al., 2020). A similar perfusion evolution could be seen in both groups, in both legs, as detected with LDF and PPG (Figure 2). Therefore, these responses were similar to the ones previously published in younger volunteers (Table 4) and consistently asymmetric, as hyperemia was always more pronounced in the massaged (test) limb than in the non-massaged (contralateral) limb. Nevertheless, a similar reflex was equally present in both age groups, as we observed no significant differences in the magnitude of these effects between age groups. In Phase III, a perfusion reduction in both age groups in both limbs was noted (Table 4), but in the aged group a significant difference persisted (*p* = 0.029).

The pending leg VAR maneuver evoked the expected significant decrease of perfusion in both legs as detected with LDF and PPG. We further compared these data with the younger participants’ values obtained under the same conditions (Figure 3) (Silva et al., 2018) and concluded that both groups’ responses were not significantly different (Table 3; Figure 3). As shown in the previous example, the response was asymmetric, being more visible in the pending (test) limb than in the contralateral limb (Table 5). Recovery also seemed to follow a similar evolution in both groups since differences between the magnitudes of these effects were not found (Table 5). Again, this procedure evoked a proportional reflex in both groups, indicating that this reflex is independent of age.

The effect of age on microcirculatory physiology remains poorly characterized, with contradictory reports on capillary density, endothelium structural modifications, and flow (Bigler et al., 2016; Groen et al., 2014). Some authors have reported a reduction of the lower leg flow with (increased) age as a consequence of less effective muscular activity and reduced oxygen demand (Bentov & Reed 2015; Dinenno et al., 1999; Donato et al., 2006; Seals 2003). Other studies assessing the influence of age during a post-occlusive response and matched-intensity leg exercise could not find differences between young and older healthy adults (Meneses et al., 2020). Recent data seem to indicate that aging impacts the entire circulatory system, as the same risk factors affect the macrocirculatory structures, reducing their buffering capacity, which in turn increases the pulsatile stress on microcirculation (Climie et al., 2019; Groenwagen et al., 2016; Huang et al., 2020; Laurent & Boutouyrie 2015). Our findings consistently identified a significant increase in peripheral perfusion in the older healthy participants. Endothelial and myogenic responsiveness could be expected in this group, even if less effective than in younger individuals. However, our results have shown that this massage hyperemia and the pending leg procedure evoked proportional reflexes that appear independent of age.

Finally, to better document and illustrate these involved mechanisms, we applied a classical PORH procedure in a group of young healthy participants, all women, and followed the circulatory responses by functional imaging (OAT-MSOT). In brief, suprasystolic occlusion (200 mmHg) was applied in the middle upper arm, on the brachial artery. After stabilization, MSOT images and videos were collected in the volar forearm for baseline. Occlusion followed (1 min) and further imaging was acquired through cuff deflation in the recovery period. The results obtained were in line with other preliminary results recently published by our group (Monteiro Rodrigues et al., 2022). As shown in Figure 4, the MSOT system allows us to visualize the two skin vascular plexuses parallel to the skin surface at different depths—larger vessels located 2–6 mm below the skin surface corresponding to the deep plexus and smaller vessels located 0.6–2 mm below the epidermis with perpendicular structures connecting both plexuses. Real-time images show (color coded) HbO2 and Hb moving from the superficial to the deeper plexus with occlusion and backward with deflation. The ROI analysis and signal post-processing provide the quantification of several variables. As shown in Figure 5, the main markers, HbO2 and Hb, change in the same direction with PORH. During occlusion, these markers practically disappear in the superficial plexus. Contraction of these vessels necessarily ensured by somatic and autonomous outflow seems to push down the blood to deeper structures. Thus, with occlusion, HbO_2_ and Hb practically disappear from the superficial plexus, increasing in the deep plexus in a similar proportion. After cuff deflation, a rapid recovery is seen in the superficial vessels. The blood pressure and pulse rate were consistently reduced along the protocol but significant differences were not detected.

These findings provide considerable evidence that sudden *in vivo* changes in local skin circulation immediately trigger this centrally mediated response to re-establish hemodynamic homeostasis. Although interventions occurred in single limb, identical responses (e.g., hyperemia increasing perfusion and VAR reducing perfusion) were noticeable in both limbs during the intervention phases. Responses seem to be proportional to the magnitude of the intervention, as shown with moderate local massage hyperemia or with the intense reduction affecting larger deep vessels evoked by the pending leg VAR or by PORH. Perfusion recovered once the intervention ceased, restoring hemodynamics. Furthermore, we have confirmed that, under these conditions, responses were similarly present independent of age and might be expected to be modified in the presence of pathological processes. Our ongoing research explores this direction further evaluating the applicability of this response as a cardiovascular marker with clinical diagnostic interest.

Our study has some limitations, such as (i) the use of a convenience sample with a reduced number of participants limits the extrapolation for the general population; (ii) the single point measurement technology chosen (LDF and PPG), although widely referenced, have recognized resolution limitations; (iii) our studies are strictly physiological, developed exclusively with healthy participants, which means that impact on these responses within specific groups of cardiovascular patients still must be established. Nevertheless, independent of the circulatory challenger or age of the participant involved, an identical adaptive response was fully reproducible. Therefore, we believe these exploratory maneuvers cannot be explained without considering a centrally mediated reflex as we have observed and documented in real time through functional imaging. Further identification of the sensors and effectors, here involved, will improve understanding of these adaptive mechanisms, potentially leading to markers with practical utility.

## Data Availability

The raw data supporting the conclusion of this article will be made available by the authors, without undue reservation.
